# Health and Disease

**Published:** 1859-11

**Authors:** 


### HEALTH AND DISEASE

Says a correspondent, "I have been confined to my bed six long years. Till within the last year I was not able to rise and walk on crutches, which is a great relief from lying on a couch from one year's end to another. I feel like a new being, for everything appears strange and new to me, as if I had opened my eyes on a new world of beauties after a long slumber. I received my first start of improved health from reading your monthly journal; for three years I have been, and am now, practising on the wholesome truths which they inculcate; *i. e.* temperance in all things; plenty of exercise in the open air, leaving off suppers entirely by persons of sedantary habits, and lessening diet one-third in spring, and during warm weather, by all classes of individuals."

The Editor is in the frequent receipt of confessions of this kind, by letter and in person, from entire strangers, from different parts of the country, and he accepts it as proof that he is living to purpose, in that he is placing the means of health in the hands of many whom he may never see, and for this, aside from any pecuniary advantage, he greatly desires that the circulation of the *Journal of Health* might be largely increased, especially in families and among young persons.

